# The Harmful Effects of Acetanilide, Antipyrin, and Phenacetin

**Published:** 1909-12-11

**Authors:** 


### Therapeutics.

### THE HARMFUL EFFECTS OF ACETANILIDE, ANTIPYRIN, AND PHENACETIN.

The Bureau of Chemistry of the United States Department of Agriculture, Washington, has recently caused a series of questions to be addressed to 925 physicians regarding the effects in their practice of acetanilide, antipyrin, and phenacetin respectively. The objects of the inquiry were similar to those of one carried out under the auspices of the British Medical Association in this country in 1894. From the replies received it is clear that the drugs in question are regarded by the profession as much more dangerous than they were formerly thought to be, with the result that the doses employed, and the number of cases in which they are applied, are much smaller. Of the three drugs, phenacetin is the most extensively used, because it is generally regarded as being less harmful than the others. The dosage is not greater than 5 grains in the case of 87 per cent. of physicians who use acetanilide, 70 per cent. of those who prescribe antipyrin, and 70 per cent. of those who use phenacetin.

### Some Tables.

The following tables may be of interest. The first is based upon the replies of the 925 physicians mentioned above, and the second upon the analysis of the cases reported in the medical literature for the period 1884 to 1907. It is somewhat curious that phenacetin, which is undoubtedly the most used, should be reported to have caused more deaths than antipyrin in the collated experience of physicians, whereas in the table drawn up from the literature it is easily last.

Table I.

| | Poisoning | Deaths | Habitual Use |
|---|---|---|---|
| Acetanilide ... | 614 | 16 | 112 |
| Antipyrin ... | 105 | 5 | 7 |
| Phenacetin ... | 95 | 7 | 17 |
| | 814 | 28 | 136 |

Table II.

| | Poisoning | Deaths | Habitual Use |
|---|---|---|---|
| Acetanilide ... | 297 | 13 | 32 |
| Antipyrin ... | 488 | 10 | — |
| Phenacetin ... | 70 | 3 | 1 |
| | 855 | 26 | 33 |

### Conclusions.

It is noteworthy that very nearly half of the cases of poisoning occurred in patients who were taking the drugs upon their own responsibility, without a doctor's prescription; and it would seem to be most important that the public should know how dangerous these remedies are unless prescribed by somebody who understands them fully. The poisoning symptoms were often produced by doses that were not greatly in excess of 5 grains, and in 85 per cent. of the cases that occurred in children toxic symptoms resulted from doses as little as 2 grains of acetanilide or of 3 grains of antipyrin.

